# Capitalizing on the uniqueness of international business: Towards a theory of place, space, and organization

**DOI:** 10.1057/s41267-022-00545-3

**Published:** 2022-07-25

**Authors:** Sjoerd Beugelsdijk

**Affiliations:** University of Groningen, Nettelbosje 2, 9700AV Groningen, The Netherlands

**Keywords:** international business, theory of the firm in space, location, business systems, othering, place, space

## Abstract

The field of international business (IB) has been successful in developing a unique body of knowledge on the multinational corporation and on country-level contexts. A recurring debate concerns its claim to uniqueness, and to associated scholarly characteristics that distinguish IB from other fields of research. I discuss what makes IB research unique by looking at what IB theory can explain and predict. To that end, I leverage key theoretical arguments and empirical insights to advance an understanding of IB centered around a firm’s ability to create added value in more than one location. I introduce a stylized model of the multi-locational firm embedded in multiple business systems characterized by equifinality. As a result of the qualitative disjunctures that separate one place from another, multi-locational firms are confronted with additional managerial and organizational challenges. These challenges are rooted in the process of “othering”. Theorizing on the critical constructs of place, space, and organization, I argue that IB offers the most generalizable approach to understanding firms doing business in more than one location. IB’s ultimate uniqueness lies in the potential of advancing a general theory of the firm in space.

## Résumé

Le domaine des affaires internationales (International Business - IB) a réussi à développer un corpus unique de connaissances sur l’entreprise multinationale et les contextes au niveau du pays. Un débat récurrent porte sur sa prétention à l'unicité et les caractéristiques scientifiques associées qui distinguent l'IB des autres domaines de recherche. Je discute de ce qui rend la recherche en IB unique en examinant ce que la théorie de l'IB peut expliquer et prévoir. Pour ce faire, je m'appuie sur des évidences empiriques et des arguments théoriques clés afin d’élaborer une meilleure compréhension de l’IB centrée sur la capacité d'une entreprise à créer de la valeur ajoutée dans plus d'une localisation. J'introduis un modèle stylisé de l'entreprise multi-localisée encastrée dans de multiples systèmes commerciaux caractérisés par l'équifinalité. En raison des disjonctions qualitatives qui séparent un lieu d'un autre, les entreprises multi-localisées sont confrontées à des défis organisationnels et managériaux supplémentaires. Ces défis sont ancrés dans le processus d'« altérisation ». M'appuyant sur les construits cardinaux de lieu, d'espace et d'organisation, j’argumente que l'IB apporte l'approche la plus généralisable pour comprendre les entreprises opérant dans plus d'une localisation. L'unicité suprême de l'IB réside dans son potentiel à faire progresser une théorie générale de l'entreprise dans l'espace.

## Resumen

El campo de negocios internacionales (IB por sus iniciales en inglés) ha venido desarrollando exitosamente un cuerpo de conocimientos único sobre la empresa multinacional y los contextos nacionales. Un debate recurrente se refiere a su pretensión de ser único y a las características académicas asociadas que distinguen a las empresas internacionales de otros campos de investigación. Discuto lo que hace que la investigación sobre negocios internacionales sea única examinando lo que la teoría de negocios internacionales puede explicar y predecir. Para esa finalidad, me apalanco en los principales argumentos teóricos y las ideas empíricas para avanzar en la comprensión de la IB centrada en la capacidad de una empresa para crear valor agregado en más de un lugar. Introduzco un modelo estilizado de empresa multilocalizada integrada en múltiples sistemas empresariales caracterizados por la equifinalidad. Como resultado de las disyuntivas cualitativas que separan un lugar de otro, las empresas multilocalizadas se enfrentan a retos adicionales de gestión y organización. Estos retos tienen su origen en el proceso de “otredad”. Teorizando sobre los conceptos críticos de lugar, espacio y organización, sostengo que negocios internacionales ofrece el enfoque más generalizable para entender a las empresas que hacen negocios en más de un lugar. La particularidad fundamental de la negocios internacionales radica en el potencial de avance de una teoría general de la empresa en el espacio.

## Resumo

A área de negócios internacionais (IB) tem tido sucesso no desenvolvimento de um corpo único de conhecimento sobre a corporação multinacional e contextos em nível de país. Um debate recorrente diz respeito à sua reivindicação de singularidade e características acadêmicas associadas que distinguem IB de outros campos de pesquisa. Discuto o que torna a pesquisa em IB única ao observar o que a teoria em IB pode explicar e prever. Para esse fim, utilizo essenciais argumentos teóricos e insights empíricos para avançar na compreensão de IB centrado na capacidade de uma empresa de criar valor agregado em mais de um local. Apresento um modelo estilizado da empresa multilocalizada incorporada em vários sistemas de negócios caracterizados pela equifinalidade. Como resultado das disjunções qualitativas que separam um lugar do outro, as empresas multilocais são confrontadas com desafios administrativos e organizacionais adicionais. Esses desafios estão enraizados no processo de ostracismo. Teorizando sobre os construtos críticos de lugar, espaço e organização, argumento que IB oferece a abordagem mais generalizável para entender empresas que fazem negócios em mais de um local. A singularidade decisiva de IB está no potencial de avançar uma teoria geral da firma no espaço.

## Abstract (zh)

国际商务 (IB) 领域成功地开发了关于跨国公司和国家层面情境的独特知识体系。一个反复出现的争论涉及其声称的独特性， 以及将 IB 与其它研究领域区分开来的相关学术特征。我通过审视 IB 理论可解释和预测的内容来讨论是什么让 IB 研究与众不同。为此， 我利用关键的理论论据和实证见解来增进对以公司在多于一个地点创造附加值的能力为中心的IB 的理解， 我提出了一个以等价性为特征的嵌入多业务系统中的多地点公司的程式化模型。由于将一个地方与另一个地方分隔开来的性质脱节， 多地点公司面临额外的管理和组织挑战。这些挑战植根于“他者化”过程。通过对场地、空间和组织的关键概念进行理论化， 我认为 IB 提供了一种最通用的方法来理解在多个地点开展业务的公司。IB 的最终独特性在于在空间发展公司通用理论的潜力。

## Introduction

There is a long tradition in international business (IB) of engaging in discussions about the added value and uniqueness of the field (Dau, Beugelsdijk, Fleury, Roth, & Zaheer, 2022). Buckley, Doh, and Benischke (2017) bring up grand research challenges, Collinson, Doz, Kostova, Liesch and Roth (2013) look at the ongoing search for universality and at the same time for context specificity, Cheng, Henisz, Roth and Swaminathan (2009) weigh in on what they see as a distinctive interdisciplinary nature, Shenkar (2004) stresses comparative research roots, and Buckley (2002) asks if the research agenda has run out of steam.

In keeping with that tradition, Aguinis and Gabriel (2022) question the uniqueness of IB. Their argument centers on complexity. They write that “organizational behavior, strategic management studies, and entrepreneurship are similarly complex because, just like in IB, theories need to consider a variety of agents and actors (i.e., multiplicity), a variety of relationships and interdependencies among these agents and actors (i.e., multiplexity), and processes as they unfold over time (i.e., dynamism in environments)” (Aguinis & Gabriel, 2022: 2). In their view, the claim of IB uniqueness is no more than a human bias rooted in the basic psychological need to be seen as special.

I think that Aguinis and Gabriel (2022) are mistaken, and that they go astray because they focus on the wrong issue. Their approach to the question of whether IB research is unique is tantamount to describing the value of cars, not by highlighting their utility but by describing differences in paint color or trim. Focusing as they do on complexity does not get us any closer to understanding for what IB theory can be used. The level of complexity of IB research may or may not be a unique characteristic, but reducing the discussion to whether IB is unique because of its complexity runs the risk of diverting the discussion away from what really matters: What is IB research good for? What can it explain?

I believe that what really needs to be addressed is what IB brings to the table in terms of better understanding firms and the contexts in which they do business. I make the case that IB is uniquely positioned to develop a “general theory of the firm in space” (Casson, 1987), and I consciously develop stylized arguments to support it. First, I lay some groundwork by describing what I see as the uniqueness of IB. In essence, the ability to theorize about the firm in its most universal conceptualization by investigating what it means to do business in other locations defines IB scholarship.

My core arguments are: (1) IB presents the most generalizable approach to understanding firms doing business in more than one location, and (2) non-IB studies are a critical step removed from a universal understanding of the firm and the way it creates added value. Most simply put, non-IB studies focus on particular cases, whereas IB studies focus on general ones. Following this train of thought, I have concluded that it is in fact non-IB studies that suffer from a uniqueness bias – the opposite of what Aguinis and Gabriel (2022) argue – and for radically different reasons.

In what follows, I will give a short summary of the historical background against which IB emerged as an institutionalized field of research.^1^ This is important, because I will argue that IB was unique from the outset. Five decades later, is the claim of uniqueness still warranted? I will make the case that the reasoning behind the original claim does not hold any longer. To describe the contemporary uniqueness of IB, I will develop in careful steps a parsimonious and comprehensive framework by adding and integrating key insights that characterize IB as a field of research, and summarize in statements the implications of the steps. Collectively, they describe what I think is unique about IB as a field of research, and I will build on that to make explicit how we can leverage that uniqueness to advance our scholarly understanding of firms and the context in which they operate.

## IB was unique, initially

The institutionalization of IB as a field of research can be traced back to the two decades following the Second World War, a period during which American firms were increasingly investing abroad (Boddewyn, 2016; Fayerweather, 1986).^2^ The economic theories that existed at the time were not able to sufficiently explain the emerging trend. Foreign capital flows were then explained by interest rate differentials between countries. It was Vernon, who led the Harvard Multinational Project from 1965 to 1973 (e.g., Vernon, 1969), and his colleagues who sparked broad scholarly interest in better understanding of why and how US firms invested abroad. Prior and also parallel to Vernon’s work in the US, Dunning studied the UK-based subsidiaries of US MNCs (Dunning, 1958). The Harvard MNC project gathered data on the development of more than 35,000 foreign subsidiaries of the world’s largest manufacturing enterprises. The material developed combined interview data, newly collected financial data, and information on modes of entry and exit, country of incorporation, and parent characteristics. Extensive manuals were produced which allowed wide-ranging access to the data (see Harvard Dataverse, Vernon, 2015).

It was around that time that Chandler (1962) published his seminal *Strategy and Structure* on the multidivisional firm, and Wilkins and Hill (1964) published their historical analysis of the international expansion of Ford Motor Company. Although Chandler (1962) did not study the multinational firm, his approach towards understanding management and the multidivisional firm by studying four firms in depth (DuPont, GM, Standard Oil and Sears) was path-breaking, and inspired others to explore to what extent Chandler’s ideas held for firms headquartered outside the US (Wilkins, 2008). Wilkins and Hill’s (1964) *American Business Abroad: Ford on Six Continents* did focus on the multinational firm and analyzed the different stages of Ford’s internationalization process between 1903 and 1963.

The data the Harvard MNC project provided was used in a number of doctoral theses on the MNC, including those of Stopford (in 1968) and Wells (in 1972), which gave birth to the renowned strategy–structure matrix (Stopford & Wells, 1972). It goes beyond the scope of this Counterpoint to discuss the genealogy of IB studies that directly or indirectly emerged from the Harvard MNC project, or other efforts intellectually related to it, such as the Reading School in the UK, but some examples are the seminal contributions of Buckley and Casson (1976), Dunning (1977, 1980), Caves (1971, 1982), Hennart (1982), and Rugman (1981).

What unites those important studies and that time period is the efforts to develop a theory of the MNC (Narula, Asmussen, Chi, & Kundu, 2019). Taking economic theory as a starting point, a key argument is that the features of the transaction explain the governance choice (market or hierarchy), and that the MNC can be explained as a mechanism to deal with uncertainty and opportunism when entering and managing foreign operations in a market (Coase, 1937; Williamson, 1975). Developing a theory of the MNC was new, because dominant theories inspired by the Heckscher–Ohlin model in international economics left little to no room for firms, let alone multinational ones, and for the way added value is created by operating across national borders. Moreover, Vernon’s studies of firms based on manager interviews was a radical departure from what at the time was considered a legitimate research design. Aharoni’s (1966) and Wilkins and Hill’s (1964) analyses of how managers make internationalization decisions were unique precisely because they focused on the inner workings of the MNC. Fayerweather, who was in 1960–1961 the first president of the Academy of International Business, was also the first to author an IB textbook, and in it he explicitly built on the notion of internal resources that can be transferred to host countries by internationalizing firms (Fayerweather, 1960), an idea that has shaped IB research ever since (Rugman & Verbeke, 1992, 2003; Teece, 2014).

That the multinational firm as an object of research was initially accorded little legitimacy is reflected in the fact that the term ‘multinational firm’ appears only 45 times in the *Journal of International Economics* throughout an entire decade following its inception in 1971, and 34 of those were in book reviews highly critical of the work of the first-generation of IB scholars. MIT economist, Bhagwati, described Vernon’s (1971) book – a summary of the Harvard Multinational Project – as ‘inadequate’ (Bhagwati, 1972: 455), and of ‘disappointing analytical quality’ (p. 456). In a review of Hennart’s (1982) book, Geroski (1984) describes the collective work of Caves, Hennart, and Buckley and Casson on the theory of the multinational enterprise as a tautology, because “this type of theorizing has a drawback in insensitive hands” (Geroski, 1984: 390).^3^

A focus on the multinational firm no longer distinguishes IB from international economics. Since Melitz’s (2003) and Bernard, Eaton, Bradford Jensen, and Kortum's (2003) successful accounts of firm heterogeneity in formal trade models, a series of international economics studies have explored the multinational firm, its organization (e.g., Bloom, Sadun, & Van Reenen, 2012), optimal entry modes (e.g., Nocke & Yeaple, 2007), and evolutionary investment paths and learning patterns (e.g., De Loecker, 2013). International economists no longer treat a firm as a subscript j or k in a model focusing on country and/or industry effects, but rather as an actor with agency (Helpman, 2006; Yeaple, 2013).

The availability of large-scale firm-level data from statistical offices has stimulated the interest of international economists in MNCs (Greenaway & Kneller, 2007) and exporting firms (Eaton, Kortum, & Kramarz, 2011). A key hypothesis and finding is that, typically, the most productive firms engage in FDI (Chen & Moore, 2010; Helpman, Melitz, & Yeaple, 2004), which bears a strong resemblance to IB theory on firm-specific advantages and the costs of doing business abroad (Rugman & Verbeke, 1992, 2003; Verbeke, 2013). International economists have also acknowledged that firms can go through a learning process when internationalizing (e.g., Schmeiser, 2012), a result that resonates with the Uppsala model of internationalization (Johanson & Vahlne, 1977), the internationalization strategy literature (e.g., Barkema & Vermeulen, 1998; Lu & Beamish, 2001), and the export development literature in international marketing (e.g. Leonidou & Katsikeas, 1996).^4^

These developments show that theories about the MNC, and location-specific and firm-specific advantages have become widely accepted in both fields. This is not to say that IB and international economics have converged after 50 years. IB theorizing on firm internationalization goes beyond generic statements on productivity or repeated exporting by unpacking the inner workings of internationalizing firms. For example, family firms may engage in FDI for family-driven reasons (Arregle, Duran, Hitt, & Van Essen, 2017), multinational firms from volatile institutional home countries may engage in foreign activity to escape the uncertain investment climate at home (Luo & Tung, 2007), and some firms may even operate abroad by mistake as a result of bounded rationality (Verbeke, Ciravegna, Lopez, & Kundu, 2019).

This brings us back to the question of whether IB is unique. Clearly it was at the beginning. The critical question is whether the claim of uniqueness is still warranted three generations later. My answer is yes, but for reasons other than that the field has focused on the MNC, which can no longer be considered a unique object of research.

**Summarizing statement 1**: Historically, IB was unique in its focus on the MNC as an object of research, but the uniqueness of IB research does not solely come from its object of research.

## IB is still unique, but for different reasons

### A Stylized Model of the Firm in Space

Let us assume a firm’s senior management thinks it can create added value by tapping into the potential of another location and leveraging its resources (broadly defined and including its managerial capabilities). That firm might, among other incentives, have market-seeking or efficiency-seeking motives, or it might be trying to obtain access to crucial resources that another firm owns, such as technology. Locational heterogeneity may, for example, provide firms with opportunities to gain from arbitrage or diversity of ideas that cultivates innovation. The focal firm becomes multi-locational by continuing to produce its product at the original location and transporting it to the target location, or by setting up a facility to produce its product in the new location, or by acquiring another firm already operating there. The key question is: How does a firm generate added value by doing business in another location? Following Dunning (2009), I see location as a key construct.

Now let us also assume that socio-economic space is homogeneous; that is, the context in which the focal firm already operates is similar to that of the target location. In that case, the only impediment is the geographic distance between the two locations. Geographic distance complicates the process of creating added value because communicating with suppliers, customers, and employees at a distance can be difficult for a firm and can raise its average costs. Yet, as long as the marginal benefits of the additional value created by doing business in a new location outweigh the marginal costs, it is attractive for the firm to operate in that location. At the very core of this thinking is location theory, as developed by the economic geographer, von Thünen (1826). In his model, the distance to market was critical in determining the location of agricultural production, a concept used by Doh (2021) to make the point that the distance construct developed in IB and location theory are fundamentally related.

The obvious disclaimer is that space is not homogenous. That assumption needs to be relaxed. The socio-economic context in which a firm operates is a continuous space with discrete moments. Put differently, the context in which a firm operates is characterized by qualitative disjunctures that distinguish one place from another (Beugelsdijk & Mudambi, 2013), and can be found at every spatial level. Cities, regions, states, clusters, countries, and supranational clusters of countries are all examples of spatial units that can (although need not necessarily) be characterized by clear border effects that distinguish insiders from outsiders (Alcácer & Delgado, 2016). For example, states may have different tax rules, cities provide agglomeration benefits not available outside them, and supranational groups of countries share legal requirements regarding trade (e.g., EU, NAFTA, ASEAN). The point is that the theoretical argument on qualitative disjunctures does not apply at just the MNC or the country level, but can in principle be generalized to all multi-locational firms and to various spatial levels of analysis.

This theoretical approach to explain the firm in space resonates with the construct of institutional fields used by institutional theorists (Greenwood, Raynard, Kodeih, Micelotta, & Lounsbury, 2011). An institutional field has been defined as “a social arena in which individuals and organizations partake of a common meaning system and interact more frequently with one another than with actors outside of the field” (Scott, 1995, as cited in Furnari, 2016). Fields are associated with specific institutional logics, defined as the overarching sets of principles that prescribe “how to interpret organizational reality, what constitutes appropriate behavior, and how to succeed” (Thornton, 2004: 70). The generalized perception that the actions of an entity (e.g., individual, firm, government) are desirable or appropriate within a socially constructed system of norms, values, and beliefs is key, because it provides legitimacy (Suchman, 1995). Organizations face the challenge of operating across multiple fields, each subject to its own isomorphic pressures, and they may be more or less compatible.

IB scholars often operationalize institutional diversity as country-specific institutional logic, either in the cross-country context of MNCs and their foreign subsidiaries or in a comparative analysis of firms across countries. In both cases, field is equated with country. Theoretically, the institutional field concept captures more than country because field boundaries are constructed around common meaning systems (Suddaby, Cooper, & Greenwood, 2007). The institutional theoretical perspective fits the general theory of the firm in space precisely because institutional logics are partly place-based and spatially bounded by the collective identification that distinguishes field members from non-members (Furnari, 2016). MNCs are not only embedded in a home and a host country, but in multiple institutional fields with varying geographical boundaries (Meyer, Mudambi, & Narula, 2011). Institutional theory and the general theory of the firm in space go hand in hand, as institutional fields are essentially relational spaces (Wooton & Hoffman, 2017).

What is critical here is the observation that certain locations have characteristics that are not found elsewhere. In other words, the space in which multi-locational firms operate is characterized by qualitative disjunctures of context. Of all the qualitative disjunctures, the one found at a country’s national borders stands out most, as the nation state (for the sake of the argument, I equate country and nation state here) is responsible for many of the relevant sources of contextual variation, including political risks (Kobrin, 1979). Administrative rules and regulations (or lack thereof), symbols and traditions, language, and norms shape the context and vary across countries. Different institutional logics can be found at various spatial levels, but are especially powerful at the country level, precisely because of coercive, normative, and mimetic pressures associated with country-specific formal and informal rules (Hofstede, 2001; Jackson & Deeg, 2008, 2019).

It goes beyond the scope of this Counterpoint to discuss thoroughly the reasons why such pressures are typically found at the country level, but key among them are the history of nation state formation,^5^ formal educational structures (e.g., school systems), the evolutionary process of the institutionalization of rules, the development of a national identity (through a shared language, symbols and heroes), and the role of international competition (sporting events, but also armed conflict) (Billig, 1995; Smith, 2000). To develop a general theory of the firm in space, it is most efficient to focus on how national borders affect the process of adding value in another location, rather than to focus on subnational or supranational qualitative disjunctures. This does not mean that regional borders cannot serve as a relevant disjuncture, but that the study of how multi-locational firms add value is best served by studying the role of national borders.

**Summarizing statement 2**: To develop a general theory of the firm in space, it is most efficient to theorize about the role of national borders and how they shape the process of multilocational firms adding value.

The key constructs of such a theory are place, space, and organization, place referring to a geographic unit of analysis not restricted to the country, and space to any characteristic that generates place variation or heterogeneity. I distinguish between the spatial transaction costs associated with continuous distance effects and with discrete border effects. Both distance and borders shape the contextual differences between the places in which firms do business, but they do so in different ways (Beugelsdijk, Mudambi, & McCann, 2010).

This is not abstract theorizing. In fact, this way of thinking has shaped recent discussion in IB on the role of global cities (e.g., Belderbos, Du, & Goerzen, 2017), the relevance of agglomeration effects at the subnational level (e.g., Ma, Delios, & Lau, 2013), the importance of the supranational regional level (e.g., Arregle, Miller, Hitt, & Beamish, 2016), and the regional (versus global) nature of geographic diversification (Rugman & Verbeke, 2004; Verbeke & Asmussen, 2016), as well as the notion of intra-country diversity (e.g., Dheer, Lenartowicz, & Peterson, 2015; Dow, Cuypers, & Ertug, 2016; Kostova & Beugelsdijk, 2021).

This generalized view of place, space, and organization encapsulates the MNC as a multilocational firm that generates added value by owning and controlling the production of goods or services in more than one country. Verbeke’s (2013) distinction between non-location and location-bound firm specific advantages to explain MNC strategies applies equally well to this general theory of the firm in space (see also Rugman & Verbeke, 1992). Similarly, Inkpen and Beamish’s (1997) argument that local knowledge acquisition affects the stability of international joint ventures can be generalized to a higher level of abstraction.

The second summarizing statement regarding national borders and the creation of added value has critical implications for the position of the MNC in IB research. By defining the object of study as the multilocational firm nested in its context, I have abstracted from the historic focus on the MNC referred to in the first statement. Taking this view still allows for considerable scope in dealing with the role of the MNC, but, by defining the higher order phenomenon of interest as the multilocational firm in context, an in-depth understanding of the MNC feeds into a more generalizable research question of how firms deal with differences in the contexts in which they do business, and how they evolve along with those. Studies of the MNC are interesting and relevant precisely because they provide an opportunity to study firm–context interaction (Kostova & Zaheer, 1999; Westney, 1993). IB is more than the study of the MNC, it is fundamentally about context and about firms that operate in more than one location, creating added value while managing the contextual differences. Moreover, comparative studies can challenge the assumptions of management theories and practices developed in one place, an observation that goes back to the work by Vernon.

### Equifinality in a Multipeak Landscape

Having established that the relationship between firm and context is best studied by using national borders as the focal qualitative disjuncture, we can theorize about the nature of cross-country differences. To understand how multilocational firms manage contextual differences, it is helpful to describe how they are organized and managed within the constraints of relatively homogenous spatial units, i.e., countries, recognizing that differences in the way firms are organized and managed are typically small within a country, and that abrupt changes occur at borders (Peterson, Søndergaard, & Kara, 2018).

Over the years, IB has developed a toolkit to effectively describe country-specific differences in organization and management. Countries develop systems of economic organization that are rooted in their history, geographic location, climate, culture, and other determinants (Redding, 2005). Those systems are not necessarily country-specific. Any number of countries may share similar determinants, for example, a common language or colonizer–colonized relationships (e.g., Great Britain and other Anglo-Saxon countries; Spain and Portugal and Latin American countries). Countries may also share certain cultural characteristics due in part to geographical closeness and historical trade patterns (e.g., the Nordics). There are two crucial issues: (1) the different aspects of such systems logically hang together and are *internally consistent,* and (2) these practices are *institutionalized* (Hall & Soskice, 2001; Scott, 1995).

The different aspects are interdependent and are related to the role of the state, the way the financial market is organized (e.g., bank-based or market-based), access to skills (e.g., the presence or absence of a vocational training system), norms and values at work (e.g., the role of hierarchy), ownership types (e.g., family firms, business groups, etc.), and labor relations (e.g., the role of unions and works councils). Different schools of thought emphasize different aspects of such systematic interdependencies in the organization and management of firms, mostly because the word ‘institutions’ takes on different meaning in different disciplines (Aguilera & Grøgaard, 2019). For the sake of keeping the overall argument about the uniqueness of IB tractable, I use the term *business system*, acknowledging that that term is associated with a specific school of thought (Whitley, 1999).

Making distinctions between business systems goes back to Albert’s (1993) contrast between the Rhineland and the North American model, and to the varieties of the capitalism approach developed by Hall and Soskice (2001). It goes beyond the scope of this Counterpoint to describe the evolutionary development of the comparative capitalism approach (Fainschmidt, Judge, Aguilera, & Smith, 2018; Witt, Kabbach de Castro, Amaeshi, Mahroum, Bohle, & Saez, 2018). I do want, however, to make the point that, over the years, IB has developed a set of robust theoretical insights into how firms in different business systems generate added value.^6^

A distinguishing feature of the comparative business systems approach is the focus on the *configurational* nature of the relationship between performance and various elements that co-determine how firms are best organized and managed in a specific location. What is ‘best’ is defined by efficiency considerations (North, 1990) and legitimacy concerns (Scott, 1995). Executives operating in different business systems will have different goals (Hofstede, Deusen van, Mueller, Charles, & the business goals network, 2002), and hold different beliefs about their responsibility vis-à-vis stakeholders, which has implications for their enactment of responsible leadership (Witt & Stahl, 2016).

The conceptualization of comparative business systems is evident in the theory of comparative corporate governance, in which systematic and institutionalized differences in the relationship between capital, labor, and management across countries inform IB research (Aguilera & Jackson, 2003). This has led to important insights regarding country-specific determinants of different types of ownership, e.g., dispersed ownership, family firms, business groups, venture capitalists, and institutional investors, and their consequences (e.g., Bruton, Filatotchev, Chahine, & Wright, 2010; Crossland & Hambrick, 2007; Filatotchev, Jackson, & Nakajima, 2013). The recognition that business systems differ shapes the literature on international finance, for example, regarding the determinants of firms’ capital structure (e.g., Kwok & Tadesse, 2006) and international accounting practices, such as earnings management (e.g., Leuz, Nanda, & Wysocki, 2003; Gray, 2006).

In essence, recognizing diversity in business systems means that IB scholars do not assume that there is a one-size-fits-all way to organize and manage a firm. No single peak exists that describes an optimal combination of different aspects of a business system, because each country’s institutional make-up is different. The space in which firms operate can thus best be described as a multipeak landscape characterized by equifinality (Fainschmidt, Witt, Aguilera, & Verbeke, 2020; Judge, Fainschmidt, & Brown III, 2014). For example, expectations of corporate social responsibility differ between Europe and the US because of their institutional differences (Doh & Guay, 2006); likewise is the difference between the Chinese and Anglo-Saxon interpretations of transaction enforcement: relationship-based (Guanxi) or rule-based (Hennart, 2015; Tsang, 1998).

Explicitly recognizing that the comparative analysis of national business systems is a core aspect of IB dates back to Vernon (1994). Shenkar (2004) goes one step further still in arguing that omitting the comparative business perspective from IB would be a fundamental error, because it is “a unique and valuable asset” (Shenkar, 2004: 164). I agree. The notion of equifinality and an understanding of the associated multipeak way firms are organized and managed across space stands in sharp contrast to the assumption that there is only one efficient and legitimate way to organize and manage firms to generate added value. The single peak model often takes western countries, specifically the US, to be the gold standard and comparative anchor (Shenkar, 2004). Although societies differ in the degree to which institutionalized practices and norms are enforced and deviations from the norm are tolerated (Gelfand, Nishii, & Raver, 2006, 2011), the observation that such differences in business systems determine the ways firms are organized and managed is a unique feature of IB research.

**Summarizing statement 3a:** IB theorizing starts from the premise that contextual differences between places are associated with different institutionalized systems of economic organization (business systems), which shape the way that firms are organized and managed through efficiency and legitimacy pressures.

**Summarizing statement 3b**: What is efficient and legitimate in one business system is not necessarily efficient and legitimate in another, and IB theorizing is informed by the foundational assumption that there is no universally most efficient and legitimate way of organizing and managing a firm.

### Here, There, and Othering

Recognizing that places, and specifically countries, differ in terms of business systems is crucial input into the general theory of the firm in space. There are challenges associated with those place-based differences, and the costs of doing business ‘here’ are not necessarily the same as doing business ‘there’. The logical theoretical implication of the existence of a ‘here’ and a ‘there’ is that of 'othering'. Firms active in multiple locations are confronted with the organizational and managerial challenges associated with othering.

Othering is related to the qualitative disjunctures that shape contextual changes and identity formation across space. What makes ‘here’ here and ‘there’ there are place-based characteristics – perceived and objective – that shape the identity of the individuals, teams, and organizations exposed to spatial differences. The result can be us versus them feelings, and insider–outsider notions that create faultlines (Stahl, Maznevski, Voigt, & Jonsen, 2010). Discrete distinctions are the strongest because of their categorical nature; nationality and religion are obvious examples. One of the reasons why the liability of foreignness is such a powerful construct in IB is that national identity is socially constructed and relational, giving meaning to ‘here’ and ‘there’ and by extension to what it means to be from ‘here’ or ‘there’.

The ontology of otherness is especially potent when based on national borders. The categorical nature of the liability of foreignness makes studies of bicultural and multicultural individuals interesting as well as relevant. The process of othering is more complex for such individuals because they identify with more than one country (Vora, Martin, Fitzsimmons, Pekerti, Lakshman, & Raheem, 2019). For them, the meaning of ‘here’ and ‘there’ is often not as unidirectional as for those born and raised and still living in a single country (Leung & Morris, 2015), which has important implications for international human resource management (Tung, 2016). This observation not only applies to bi-cultural and multicultural individuals but also to expatriates. For example, Fan and Harzing (2017) show that the way host-country employees and expatriates ethnically identify affects cooperation between local and expatriate employees. The language spoken is also a discrete category that generates faultlines and that matters especially in the context of IB (Tenzer, Pudelko, & Harzing, 2014; Vaara, Tienari, Piekkari, & Säntti, 2005).

IB scholars have extensively studied the challenges associated with othering. The concept of being (or perceived to be) different is fundamental to IB research in terms of how firms deal with cross-national differences or benefit from them. Following the business systems logic, cross-national differences are multidimensional and related to cultural, institutional, political, and economic aspects of context, as well as to perceptions of those differences (Beckermann, 1956; Dow & Karunaratna, 2006). ‘Here’, ‘there’ and 'othering' are at the core of the integration–responsiveness framework, giving shape to the tension between global pressure for standardization and local pressure for adaptation (Prahalad & Doz, 1987), as well as country-of-origin effects in consumer preferences (e.g. Balabanis & Diamantopoulos, 2004) and home and foreign biases in financial decision making (e.g. Chan, Covrig, & Ng, 2005). The MNC is typically portrayed as a boundary-spanning firm balancing challenges associated with multiple embeddedness (Schotter, Mudambi, Doz, & Gaur, 2017) and this has important implications for international strategy (e.g., Brouthers, 2002) and international marketing (e.g., Theodosiou & Leonidou, 2003).

The simple von Thünen model, to which I alluded earlier, in which geographic distance between two locations is assumed to determine the ability to generate added value in another location, has been enriched by adopting a much broader notion of distance (Berry, Guillen, & Zhou, 2010). The attractiveness of the distance construct rests on its literal meaning of geographic distance, as well as on its metaphorical one (Shenkar, 2012), which refers to “the collective differences between countries” (Zaheer, Schomaker, & Nachum, 2012: 20). It is fair to say that the cross-national distance construct – just like the related construct of friction (Shenkar, Luo, & Yeheskel, 2008) – has become a key feature of IB research (for reviews, see Beugelsdijk, Kostova, et al., 2018; Beugelsdijk, Ambos, et al., 2018; and Kostova, Beugelsdijk, Scott, Kunst, Chua, & Essen van, 2020).

Although the distance framework is dominated by a cross-country approach, its underlying theoretical argumentation on the costs of doing business in other contexts can be extended to other spatial levels of analysis. Verbeke and Asmussen (2016) studied MNC strategies and the relevance of supra-national regions, and argued that “outsiders to the region face a compounded distance ‘spike’ relative to the insiders” (p. 1056). At the subnational level, Gao, Wang and Che (2018) have shown that Japanese investment in China is negatively affected by the civilian casualties suffered in different provinces of China during the second Sino-Japanese war, demonstrating that historical animosity can feed into contemporary processes of othering.

A number of IB scholars have analyzed the managerial challenges associated with the transfer of practices from ‘here’ to ‘there’ (Kostova, 1999; Kostova & Roth, 2002). Rooted in Meyer and Rowan’s (1977) observation that contextual differences may result in incompatible pressures, the MNC is *the* example of a firm that has to manage “incompatible prescriptions from multiple institutional logics” (Greenwood et al, 2011: 317), especially when transferring capabilities across markets and countries (Foss & Pedersen, 2002; Kogut & Zander, 1993). Practices considered legitimate and/or efficient in one context may not be considered to be so in another (Fiss & Zajac, 2004). For example, the German institutionalized tradition of apprenticeship-based training does not exist in the US, and this has posed significant human resources difficulties for BMW in setting up a factory there (Fortwengel, 2017). MNCs can buffer negative consequences of othering at lower levels (Fortwengel, 2021). For instance, an MNC with a strong overarching organizational identity can reduce the negative effects of faultlines between units and teams, and within teams, as typified by “We all work for Google”. The successful transfer of practices is also related to the meaning of foreignness and the interpersonal dynamics between transmission and reception, a process also influenced by language (Brannen, 2004).

**Summarizing statement 4a:** IB research has shown that, when multilocational firms engage in purposive action to create added value outside of their own business system, they need to balance pressures for efficiency and/or legitimacy in their home business system with those for efficiency and/or legitimacy in the ‘host’ business system.

**Summarizing statement 4b:** As a result of perceived and objective differences between ‘here’ and ’there’, processes of othering affect the overall performance of groups of individuals in firms and organizational units of firms doing business in more than one location compared to otherwise similar firms doing business in a single location.

### Roots of Stability and Forces of Change

The general theory of the firm in space is dynamic. The ‘here’ and the ‘there’ change, as does the nature of othering. There are two opposing forces. Business systems tend to be stable because practices are institutionalized, creating path dependency and predictability. The dynamics come from endogenous changes and exogenous shocks. US firms operating there today do so in a context different from that of three decades ago, just as are those doing business in China now compared to 30 years ago. A theory of the firm in space needs to account for such change. IB scholars are in a good position to do that, having studied those dynamic aspects extensively, as well as their impact on firm strategy (Doh & Lucea, 2015), especially in the context of emerging markets (e.g., Hoskisson, Eden, Lau, & Wright, 2000; Meyer, Estrin, Bhaumik, & Peng, 2009; Park, Li, & Tse, 2006).

Change is multidimensional. First, the socio-economic context in which firms operate changes, often as a result of technological progress (Luo, 2022). Second, political ideologies change. After decades of neoliberal policies in western countries, as exemplified by the administrations of Prime Minister Thatcher in the UK and President Reagan in the US, the pendulum is swinging in another direction, although the extent to which is still unclear, as are the implications for globalization (Witt, 2019). While the Washington consensus shaped much of the debate about the preferred economic organization of countries in the 1980s and 1990s, today that seems almost archaic. The same holds true for terms like “Asian tigers” to describe the rapid industrialization of South Korea, Taiwan, Singapore, and Hong Kong between the 1960s and 1990s. Contemporary political ideological discussions instead revolve around populism and its implications for global strategy (Devinney & Hartwell, 2020). More generally, pro-market reforms and reversals bring about change in the institutional contexts in which firms operate (Cuervo-Cazurra, Gaur, & Singh, 2019), and institutional transition has important implications for firm strategies (Park et al., 2006). Third, in the long run, all societies undergo cultural change. As a result of generational replacement, many societies have generally become more individualistic, less hierarchical, and more focused on the short term (Beugelsdijk & Welzel, 2018), with important implications for firms, for example, in terms of HRM practices. All these changes, cultural, political, institutional, technological, and economic, are interdependent; for instance, economic progress has led to change in values (Inglehart, 1997), and structural change in an economy often leads to a reshuffling of winners and losers and political change.

Change does not only come from the outside, firms themselves shape the context in which they do business, and MNCs especially have the ability to do so. MNCs are often large firms, and have more highly educated employees compared to domestic firms (van der Straaten, Pisani, & Kolk, 2019). In addition, they tend to be politically well connected, and can also leverage resources from various countries (Sun, Doh, Rajwani, & Siegel, 2021). Firms have agency in shaping the context in which they do business, both at home and abroad, as exemplified by research on corporate political activity (e.g., Holburn & Zelner, 2010), stakeholder management (e.g., Nartey, Henisz, & Dorobantu, 2018), and non-market strategies (e.g., Doh & Lucea, 2015).

Although clear examples of exogenous shocks do exist (e.g., political coups and other forms of revolutionary political change, and supposedly once-in-a-lifetime events like the 2008/2009 financial crisis and the Covid-19 pandemic), the nature of contextual change is often a combination of exogenous and endogenous developments. For example, the opening up of China led to a dramatic increase in offshoring activity by western firms, raising wages in China, and sparking a positive learning curve for local Chinese firms, as well as changes in the competitive landscape for offshoring western firms. The relationship between FDI and economic development is bidirectional, but the exact nature of the relationship changes, depending on the location-specific advantages that MNCs can tap into and the ownership advantages that they have (Narula & Dunning, 2000). The relationship between firms and the changing contexts in which they do business is best described as co-evolutionary (Cantwell, Dunning, & Lundan, 2010; Lewin, Long, & Carroll, 1999).

Integrating dynamics in the theory of the firm in space has two obvious implications. First, business systems change has consequences for firms. Second, the nature of doing business *across* business systems also changes, because change affects the nature of ‘othering’ rooted in objective and perceived differences between business systems (Kobrin, 2019). It is not simply a matter of convergence or divergence of business systems and associated ideologies (Ralston, Holt, Terpstra, & Kai-Cheng, 1997). Some dimensions of cross-national differences diverge, others converge, and some develop in parallel directions (Berry et al., 2010; Beugelsdijk, van Hoorn & Maseland, 2015; Taras, Steel, & Kirkman, 2012).

Contextual changes in the socio-political and economic environment make salient the challenges associated with othering (Kobrin, 2019). We see this manifested in the current political climate of rising nationalism, which feeds into self-identification along country lines, but also in intra-country divisions along religious, racial, ethnic, and political ultra-left and ultra-right lines. The way in which people identify amplifies discrete qualitative disjunctures and faultlines on the shop floor. For example, it may be less a case of change in cross-cultural value differences than in the extent to which those differences matter. This underscores the need to distinguish between contextual changes of a continuous nature (e.g., differences in cultural values), and changes of a discrete nature (e.g., the meaning of nationality). As the latter have a stronger effect on the perceptual and non-perceptual differences between ‘here’ and ‘there’, change is not simply a continuous evolutionary process in which what happens today is the result of an extrapolation of the past (Cantwell et al., 2010).

**Summarizing statement 5a**: Exogenous shocks make the business system in which firms operate dynamic, which changes the way added value is created ‘here’ and ‘there’, as well as the organizational and managerial challenges associated with the process of othering.

**Summarizing statement 5b**: The nature of contextual change is co-evolutionary, because firms, especially large ones, that do business in multiple locations can shape the contexts in which they operate.

IB is well placed to address the changing nature of the relationship between firms and the context in which they operate, precisely because many of the grand challenges that firms are confronted with deal with contextual change (George, Howard-Grenville, Joshi, & Tihanyi, 2016; Van Assche & Lundan, 2020). For example, how do exogenous shocks like the Fukushima nuclear disaster, Covid-19, and wars in Syria, Ukraine, and Yemen impact the world economy and the strategic response of firms across different countries? How is the rise of nationalism and the return of the state related to firm internationalization strategies? What is the role of firms in addressing societal polarization, and intensified feelings of us versus them, affecting the meaning of “here” and “there” (Ganson, He, & Henisz, 2022)? And how does the spatial variation in the consequences of climate change affect firm strategies across different regions in the world? It is these kinds of important questions that require a deep knowledge of both firms and the context in which they operate.

## Conclusions

### Where I Differ with Aguinis and Gabriel (2022)

A discussion on a topic as broad as the uniqueness of a field of research is bound to be incomplete. It is impossible to do justice to all the scholarly insights developed in the literature, especially for the IB field that is characterized by theoretical pluralism. Moreover, many of those insights have appeared in *JIBS*, but many of the ones on which IB scholars build date back further than the establishment of this scholarly journal in 1970. Given that, addressing IB uniqueness becomes a risky endeavor because incompleteness opens the door to a strawman view of what makes IB unique. An engaged discussion on the domain of IB of the kind I have undertaken could even possibly be misinterpreted as a signal that IB scholars themselves do not agree on what the field is about. A more constructive interpretation of the willingness to engage in discussions of this kind is that, as a community, IB scholars have institutionalized open debate on the nature of the questions that should be asked, the boundaries of the field, the most effective theories to explain the phenomena of interest, and the preferred methodological tools to assess their predictive power.

Aguinis and Gabriel (2022) carry on the IB tradition of questioning the uniqueness of IB as a field of research. In their view, it is false to claim that IB is unique, and to do so is no more than a manifestation of a basic psychological need to be seen as special. I do not find their point compelling, because their arguments also apply to non-IB fields. Scholars in the fields of organizational behavior, strategic management, and entrepreneurship (the three fields mentioned by Aguinis and Gabriel) also find it easier to access information in their respective fields compared to other fields (what Aguinis and Gabriel call selective accessibility), and give more weight to information that is readily available (what they call focalism). In any situation of information overload and high search costs, cognitive sensemaking is associated with selective accessibility. Add to this the socio-psychological mechanisms aptly described by structuration and diversity theorists (Byrne, 1971; Giddens, 1984; Tajfel, 1981, 1982) and the result is a community of scholars defining themselves, not only on the basis of what they do but also what others do. Compartmentalization and development of scholarly identities is not unique to IB.^7^ The collective outcome of individual decision-making by groups of scholars is one that actually is fundamentally rooted in human behavior.

What is more important though is the focus of Aguinis and Gabriel (2022). The relevant question to ask is not whether complexity characterizes IB but for what IB theory can be used. This is what I have done in this Counterpoint. I do not believe the uniqueness claim that characterizes IB is false. I have described how IB was different at inception, and shown that the uniqueness claim that Aguinis and Gabriel (2022) call false is in fact not. IB was on the defensive in the early years precisely because it was different, and this shaped the field and the scholars in it.^8^ Yet, that past cannot be seen as a prologue because arguments that applied decades ago no longer apply today. Instead, I have argued that IB’s contemporary uniqueness is derived from the opportunities that its research provides to advance a theory of the firm in space.

### Proximate and Ultimate Uniqueness of IB

The challenge for IB is to lift the theoretical insights generated by studying internationalizing firms and MNCs to a higher abstraction level in order to arrive at such a theory of the firm in space (Roth & Kostova, 2003). To that end, I distinguish between the proximate and ultimate uniqueness of IB. By proximate uniqueness, I mean advances in the theory of the MNC and the internationalizing firm, the store of knowledge IB scholars have generated about country-level contexts and business systems in which firms across the world operate, and also the improved understanding of the co-evolutionary relationship between the MNC and these changing contexts. The ultimate uniqueness of IB lies in a theory of the firm in space of which I have laid out key characteristics in previous sections. IB has been very successful in establishing its proximate uniqueness, but less so in explicating its ultimate uniqueness.

One way to demonstrate the proximate uniqueness of IB is to list all the practical questions that would have remained unaddressed without IB research. Thanks to five decades of IB research, we understand why firms invest abroad and which entry mode will be preferred, depending on the characteristics of the transaction, of the firm, and of its host-country partner(s). We know how the internationalization process of SMEs differs from that of large established firms. We understand why it is so difficult for firms to transfer their practices from the home country to a subsidiary in a host country, and how to address the transfer challenges. By analyzing the experiences of bicultural and multicultural individuals and expatriates, IB has set out how human resource practices can be adapted to create added value in a host country. Extensive analyses of headquarters and subsidiaries have provided tools which allow firms to optimize knowledge flows between units. We have delineated the pros and cons of FDI data, performance measures, and indicators of geographic diversification of internationalizing firms. Last, but by no means least, the debate centered on the nature of emerging market MNCs has yielded a much better understanding of the differences and commonalities between them and their developed country counterparts.

I decided against organizing my discussion of IB’s uniqueness using a list of such collective achievements, as it is simply impossible to provide an exhaustive one. Instead, I have framed the uniqueness of IB in terms of its ability to address how firms generate added value by doing business in other locations. IB is not special because it is a unique subfield of business studies or of general management. I believe that it is unique because its theoretical assumptions best reflect the conditions under which the most generalizable theory of the firm in space can potentially be developed. By relaxing the assumption that contextual differences matter for firms, non-IB studies are a critical step short of such a general theory of the firm in space, even though they have relevance to the extent that they fit into the bigger picture of the multipeak landscape characterized by equifinality. The challenge for IB and non-IB scholars alike is to leverage the insights IB has generated to further develop a general theory of the firm in space. This is not obvious, as it would require that IB scholars also invest in going beyond the “narrow” international aspect of MNCs, and that non IB-scholars would acknowledge the presence of contextual variation as a foundational assumption guiding all their work.

Let us acknowledge that scholars, just like firms, operate in a particular context. Theorizing does not take place in a vacuum. The way we theorize about the firm is shaped by our backgrounds because of our cognitive limitations, socialization in a particular setting, and cultural and institutional pressures (Hofstede, 1983). Management research is dominated by theoretical perspectives that originate in North America and Europe. To counter that, Filatotchev, Duane Ireland and Stahl (2021) propose what they call an open systems perspective to “draw attention to the broader context in which organizations are embedded” (p. 2). Their suggestion fits a recent trend of questioning the under-contextualized nature of mainstream management research (Aguinis, Villamor, Brutton, Lazzarini, Vassolo, Amorós, & Allen, 2020; Barkema, Chen, George, Luo, & Tsui, 2015; Brutton, Zahra, Van de Ven, & Hitt, 2021).

As much as I can appreciate the importance of context-sensitivity, I must object to this most recent debate on the importance of context. My reasoning is two-fold. First, by using terms such as *indigenous theorizing* (Bruton et al., 2021), we effectively re-confirm western theories as being *the* scholarly anchor (Tsui, 2018), a persistent phenomenon that resembles Said’s orientalism (1978). Although Van de Ven, Meyer and Jing (2018) carefully discuss what they mean when they use it and acknowledge its possible neo-colonialist connotation, Adler’s (1983) classic distinction between idiographic and nomothetic research approaches is more neutral and thus preferable. Second, IB scholars also have a long tradition of debating the tension between universality and context-specificity of theoretical frameworks. The fact that general management increasingly recognizes the relevance of context underscores my general point that IB is in fact the general theory. Let me conclude by summarizing the arguments I have developed here in the most succinct, some might say extreme, way: what we commonly refer to as general management theory may just have been about the special case, and what is often called a special case of general management (i.e., the field of IB) may in fact be closer to a general theory of the firm in space. That is precisely what makes IB unique.

## NOTES

1. The institutionalization of the IB field is not the same as a description of its intellectual heritage. As Shenkar (2004) describes in his assessment of what makes IB unique, many of the scholarly insights on which IB scholars have built date back to long before the field was institutionalized as reflected in the establishment of a scholarly association (AIB was established in 1959 starting with 19 members) and a journal (the first issue of *JIBS* was published in 1970).
2. Multinationals did exist before WW2. Wilkins (1974) shows that the international expansion of US firms is not just a post-WW2 phenomenon, rather there is a long history which runs parallel to the general growth pattern of US business. Similarly, Jones (2005) describes globalization waves in the nineteenth and twentieth centuries. My point is that professional scholarly interest in IB took off in the first two decades after WW2 which coincides with the increasing presence of US MNCs worldwide. As it was put by Terpstra in the very first article published in the *JIBS*, “From all evidence that can be gleaned from this study and previous inquiries, university education for international business is indeed a ‘going thing’. One reason is undoubtedly the belated recognition by American business schools that internationalism is coming to be a way of life for American business. New business school graduates with a long career ahead of them must be ready for this internationalism” (Terpstra, 1970, volume 1, issue 1, page 96).
3. There is a well-known anecdote about Hymer’s PhD thesis which illustrates the initial lack of scholarly legitimacy accorded the study of the MNC: Hymer’s 1960 dissertation, entitled *The international operations of domestic firms*, presented a Coasian analysis of FDI and the MNC (Dunning & Rugman, 1985). The question of why firms internalize was applied to the MNC, and today Hymer’s analysis of the monopolistic MNC is generally considered to be a classic (for overviews of Hymer’s work, see Dunning and Pitelis (2008) and Pitelis (2006), and for a discussion of the school of thought on the basis of which Hymer developed his framework, see Buckley (2011). Although publication of theses was common at the time at MIT, Hymer’s dissertation was not published because, as one member of the committee put it, “the argument was too simple and straightforward” (Pitelis, 2002: 10). The thesis was eventually published, but posthumously in 1976. In the foreword, Hymer’s thesis supervisor Kindleberger writes about the thesis enjoying “a notoriety and underground existence” (Kindleberger 1976, p. xiv, cited in Pitelis, 2002: 9). That a seminal work has been treated with such disregard is a powerful illustration of the lack of legitimacy of IB in the early years, and ironically has contributed to the cult status of Hymer (Pitelis, 2002).
4. Despite a shared interest in the MNC, the conceptual overlap with respect to the critical role of firm specific resources, and an increased use of firm-level data in international economics, IB scholars and international economists continue to operate largely isolated from one another (Beugelsdijk, van Ees, Garretsen, & Brakman, 2013). It is telling that, up until June 2, 2021, the total number of cross-citations between the two flagship journals – *Journal of International Business Studies* (*JIBS*) and *Journal of International Economics* (*JIE*) – is only 266, less than .5 percent of the citation combined total since their respective inceptions, 1970 in the case of *JIBS* and 1971 for *JIE*, (Web of Science; 2021). Work appearing in *JIBS* is cited in *JIE* 34 times since 1970, and work appearing in *JIE* 232 times in *JIBS.* That only two of the citations in *JIE* were in the 1970s, and almost half of the remainder in the last decade alone, underscores the initial lack of legitimacy accorded the study of the multinational firm.

5 For example, the Treaty of Westphalia in 1648 for Europe and the Treaty of Chanyuan in 1005 for China have been pivotal in the process of nation-state formation.
6 Although Chandler did not develop the institutional argument to the extent that this has been done in the business systems literature, the basic idea that large private firms operate in a context which shapes their evolution has been an important element of much of Chandler’s work since he published *Strategy and Structure* in 1962 (Wilkins, 2008). For example, Chandler (1990) discusses comparative business histories of US, UK, and German firms, and Chandler, Amatori and Hikino (1997) do so for firms across 19 countries.
7 During the process of the studying of the inner workings of the MNC, not only were language, heroes, and keywords specific to IB developed, but the basis of the field was expanded. While the intellectual heritage of the Coasian understanding of the firm remained solid, as reflected in the dominance of transaction costs theory in IB, the disciplinary sources of inspiration broadened. Eden (2008) has referred to IB as a row discipline with other disciplines (marketing, finance, etc.) in the columns. Yet, the multidisciplinarity of IB is not what makes it unique as a field of research. Just as it can be argued that complexity also characterizes other fields, so is the case with multidisciplinarity, which also characterizes entrepreneurship studies and strategic management.
8 The perceived need to explain and even ‘defend’ IB’s relevance has become part of its intellectual DNA, and may have instilled a culture that has been passed on from the first generation to the next. It is impossible to test, but I would conjecture that one plausible reason for the recurring uniqueness discussion in IB can be traced back to the origin of the field as a spin-off of international economics, and as a field that lacked legitimacy at the time because of the focus on the multinational firm and the research methods employed.
